# *Pla2g12b* and *Hpn* Are Genes Identified by Mouse ENU Mutagenesis That Affect HDL Cholesterol

**DOI:** 10.1371/journal.pone.0043139

**Published:** 2012-08-17

**Authors:** Aleksandra Aljakna, Seungbum Choi, Holly Savage, Rachael Hageman Blair, Tongjun Gu, Karen L. Svenson, Gary A. Churchill, Matt Hibbs, Ron Korstanje

**Affiliations:** The Jackson Laboratory, Bar Harbor, Maine, United States of America; Klinikum rechts der Isar der TU München, Germany

## Abstract

Despite considerable progress understanding genes that affect the HDL particle, its function, and cholesterol content, genes identified to date explain only a small percentage of the genetic variation. We used *N*-ethyl-*N*-nitrosourea mutagenesis in mice to discover novel genes that affect HDL cholesterol levels. Two mutant lines (Hlb218 and Hlb320) with low HDL cholesterol levels were established. Causal mutations in these lines were mapped using linkage analysis: for line Hlb218 within a 12 Mbp region on Chr 10; and for line Hlb320 within a 21 Mbp region on Chr 7. High-throughput sequencing of Hlb218 liver RNA identified a mutation in *Pla2g12b*. The transition of G to A leads to a cysteine to tyrosine change and most likely causes a loss of a disulfide bridge. Microarray analysis of Hlb320 liver RNA showed a 7-fold downregulation of *Hpn*; sequencing identified a mutation in the 3′ splice site of exon 8. Northern blot confirmed lower mRNA expression level in Hlb320 and did not show a difference in splicing, suggesting that the mutation only affects the splicing rate. In addition to affecting HDL cholesterol, the mutated genes also lead to reduction in serum non-HDL cholesterol and triglyceride levels. Despite low HDL cholesterol levels, the mice from both mutant lines show similar atherosclerotic lesion sizes compared to control mice. These new mutant mouse models are valuable tools to further study the role of these genes, their affect on HDL cholesterol levels, and metabolism.

## Introduction

Over the past few decades the incidence of cardiovascular diseases, caused by underlying atherosclerosis, has increased and become a public health concern [1], [2], [3], [4]. HDL cholesterol level is a negative risk factor for atherosclerosis and raising its level has been identified as a preventative strategy for disease management [5], [6], [7]. Despite considerable progress that has been made through genetic associations and studies on model organisms to unravel regulation of the HDL particle and its cholesterol content, recent studies suggest that gaps in knowledge about HDL regulation and its role in the disease remain to be filled [8]. First, genes identified to date explain only a small percentage of genetic variation, suggesting that many genes are yet to be identified [9]. Second, several clinical studies have identified individuals with a significant atherosclerosis burden despite low, normal, or elevated levels of HDL cholesterol [7], [10]. Third, although torcetrapib trials demonstrated significant increase in HDL cholesterol levels, the study failed to show a reduction in cardiovascular events [10]. Knowing and understanding genes that affect the HDL cholesterol, function, and protein content in full detail is critical: It will help us understand its role in lipid metabolism and in the development of atherosclerosis, and predict unwanted side effects of future treatment [6], [8]. We aim to discover novel genes that contribute to the phenotypic variability of HDL cholesterol levels.

One approach for identifying novel genes is by *N*-ethyl-*N*-nitrosourea (ENU) mutagenesis in mice [11], [12]. Genes identified through this approach would either directly affect the HDL particle, its cholesterol content, or both, or indirectly influence metabolites and metabolic pathways that in turn affect the HDL particle, its cholesterol content, or both. Using this approach we established 2 mouse lines (Hlb218 and Hlb320) with low HDL cholesterol, identified the causal mutations, and characterized the mutants.

## Materials and Methods

### Animals, Housing, and Diet

Mutant mice (G0) were generated as part of The Jackson Laboratory's Heart, Lung, Blood, and Sleep Disorder Mutagenesis Program by treating male C57BL/6J (B6) mice with *N*-ethyl-*N*-nitrosourea (ENU). Protocols for generating, phenotyping and heritability testing of these ENU lines were described previously [12]. Briefly, to capture both dominant and recessive mutations, G0 mice were backcrossed twice to the B6 strain to generate G2 mice, which were then backcrossed to G1 mice to generate third generation ENU mutants (G3). Phenotyping G3 progeny identified two unique G3 animals with low HDL cholesterol levels that were then used to establish new inbred lines (Hlb218 and Hlb320): first, G3 (N2F1) animals were backcrossed to B6 mice (the third backcross); then their progeny (N3F1) with the low HDL cholesterol phenotype were further intercrossed (the number of intercross generations varied by experiment; see materials and methods section of each experiment for generation information). Both lines were cryopreserved and are publically available. The Mouse Genome Database (www.informatics.jax.org) accession numbers and JAX® Mice database (http://jaxmice.jax.org) stock numbers are as follows: Hlb218—MGI:2678708, stock #008508; Hlb320—MGI:3575147, stock #008507. C57L/J (C57L) and C57BL/6J-*Ldlr^Hlb301^*/J (*Ldlr* ENU) mice were purchased from The Jackson Laboratory, Bar Harbor, ME. All mice were housed in a temperature- and humidity-controlled pathogen-free facility with a 12 h∶12 h light∶dark cycle. Mice were housed in pressurized, individually ventilated duplex cages on shaved pine bedding and had free access to acidified water and a standard rodent chow diet containing 6% fat by weight (5kK2 LabDiet, Brentwood, MO). Mice involved in the test for susceptibility to atherosclerosis were fed an atherogenic diet (18.5% dietary fat, 1.9% corn oil, 50% sucrose, 4.1% cellulose, 20% casein, 1% cholesterol, 0.5% cholic acid, 5% mineral mix, 1% vitamin mix, 0.3% DL-methione, 0.13% α-tocopherol, 1% choline chloride; similar to the previously described diet) [13]. All experiments were approved by The Jackson Laboratory's Animal Care and Use Committee.

### Genetic Mapping and Linkage Analysis

To map the ENU mutations to a chromosomal position, linkage analyses on (ENUxC57L) F2 mice were performed. The C57L strain was chosen as a mapping strain because, while it provided enough polymorphisms to perform genetic mapping, its genetic proximity to the background strain of the ENU mutants (B6) reduced the presence of HDL cholesterol quantitative trait loci (QTL) caused by natural polymorphisms between the two strains. Briefly, mutant mice (Hlb218 generation — N3F5; Hlb320 generation — N3F8) were crossed with C57L mice, and F1 offspring were intercrossed to generate 81 Hlb218 and 75 Hlb320 F2 progeny, which were phenotyped at 8 weeks of age for plasma HDL cholesterol levels as described below. DNA from each F2 mouse was extracted from the tail tip, isolated by phenol∶chloroform extraction, and genotyped by KBiosciences, Herts, UK for 58 (Hlb218) and 61 (Hlb320) single-nucleotide markers (polymorphic between B6 and C57L) that cover the complete genome (http://cgd.jax.org/cgdsnpdb). Linkage analysis was performed using interval mapping methods specific for a binary trait within the R/QTL package (R version 2.8.0, qtl version 1.09–43). Mice that exhibited plasma HDL cholesterol levels similar to B6 and C57L were considered not affected and were coded 0. Mice with plasma HDL cholesterol levels that were two standard deviations below the mean of normal B6 mice (≤40.2 mg/dL for females and ≤50.0 mg/dL for males) were considered affected and were coded 1. A genome-wide scan was done with 1,000 permutations. The significant LOD score threshold was calculated by permutation testing at α = 0.05 [14]. For (Hlb218×C57L)F2 mice the threshold LOD score was 3.44, and for (Hlb320×C57L)F2 mice it was 3.46. The mode of inheritance of the allele was determined by performing a one-way ANOVA using the effect plot function within the R/QTL package and confirmed by Tukey-Kramer HSD: animals were grouped by genotype and sex, and the average HDL cholesterol level of each group was compared [15]. Once a chromosomal position was identified, affected animals with crossovers on that chromosome were genotyped with additional polymorphic markers to narrow the interval.

### Analysis of HDL Cholesterol, Total Cholesterol, Triglyceride, Alkaline Phosphatase, and Thyroxine

Blood was collected via retro-orbital sinus from animals that were food-deprived for 4 hours in the morning. Blood intended for preparation of plasma was collected into tubes containing EDTA. Plasma and serum were separated by centrifugation (14,000 rpm for 10 minutes in an Eppendorf Centrifuge 5424 with rotor FA-45-24-11 [20,238×g/14,860 rpm]) and frozen at −20°C until analyzed. Plasma and serum samples were analyzed for lipid, total alkaline phosphatase, and thyroxine levels on the Beckman Coulter Synchron CX®5 Delta autoanalyzer (Beckman Coulter, Inc., Brea, CA) within one week of collection date (HDL cholesterol: enzymatic reagent kit #650207; total cholesterol: enzymatic reagent kit #467825; triglycerides: enzymatic reagent kit #445850; total alkaline phosphatase: enzymatic reagent kit #442670; thyroxine: enzymatic reagent kit #445995). Serum lipid levels: Hlb218 generation — N3F8; Hlb320 generation — N3F11. Alkaline phosphatase and thyroxine levels: Hlb320 generation — N3F9.

### Microarray and RNAseq Analysis

Livers from 3 Hlb218 (N3F4), 3 Hlb320 (N3F5), and 6 B6 males were obtained for gene expression analysis (microarray and RNAseq). All males were 8 weeks old. Prior to tissue collection, males were housed individually for 3 days, food-deprived for 4 hours (7 am to 11 am) on the day of tissue collection, sacrificed by cervical dislocation, and perfused using DEPC treated 0.9% NaCl solution. The liver samples were stored in RNAlater (Ambion, Austin, TX) and homogenized in TRIzol™ (Invitrogen, Carlsbad, CA). Total RNA was isolated by TRIzol™ Plus methods according to the manufacturer's protocols. RNA quality was assessed using an Agilent 2100 Bioanalyzer instrument and RNA 6000 Nano LabChip assay (Agilent Technologies, Palo Alto, CA).

For microarray analysis, RNA was prepared using an Illumina® Totalprep RNA amplification kit according to the manufacturer's protocol (Ambion, Austin, TX). Liver RNA samples were hybridized on Illumina Mouse-6 Expression 1.1 BeadChips (Illumina, San Diego, CA) using the Illumina BeadStation 500× followed by statistical analysis of the data. Probe set data (mean pixel intensities by bead type) were created using BeadStudio (version 3.0.19.0) and processed using the R/beadarray package [16], [17]. The data were log-transformed and normalized [18]. ANOVA models were used to determine gene expression differences between each mutant strain and the B6 controls [19]. Statistical tests were performed using a modified F-statistic that incorporates shrinkage estimates of variance components [20]. P-values were calculated by permuting model residuals 1,000 times. Calculations were done using the R/maanova package. To identify candidate genes in the mapped interval, statistical significance was calculated using Bonferroni correction: correction was applied to a subset of genes in the mapped interval to account for multiple testing. To identify all of the significantly differentiated genes between Hlb320 and B6, the false discovery rate (FDR) was estimated using a q-value calculation for the set of statistically significant probes [21]. Gene expression data is available through Gene Expression Omnibus (GEO) Accession GSE37902.

For RNAseq, the NEBNext mRNA Sample Prep Master Mix Set I kit (New England Biolabs, Inc., Ipswich, MA) was used to prepare the sequencing libraries. These libraries were sequenced single-end on an Illumina HiSeq 2000 instrument (Illumina, San Diego, CA). Every read was aligned to the NCBI mouse reference genome (mm9) using the Bowtie alignment software tolerating 2 mismatches [22]. Mismatches with high base quality scores that occurred only in the unique mapping of a read to the genome were considered potential SNP sites. SNPs were called at sites where the percentage of reads containing the apparent SNP were at least 90% of all reads mapped to the site, and where at least 5 high quality score reads were present. Finally, SNPs were annotated based on known SNPs from UCSC (http://genome.ucsc.edu), dbSNP (http://www.ncbi.nlm.nih.gov/projects/SNP/), and the Center for Genome Dynamics SNP database (http://cgd.jax.org/cgdsnpdb/). SNPs were further confirmed by Sanger sequencing.

### Sanger Sequencing

The mutation in Hlb218 identified by RNAseq was confirmed by Sanger Sequencing. The third exon of *Pla2g12b* was amplified using genomic DNA from Hlb218 and B6 mice. Conservation of the mutated cysteine was assessed by evaluating the sequence of exon 3 in 12 mammals (Ensembl accessed in May 2012). The mutation in Hlb320 was identified by comparing the sequences of all *Hpn* exons, including the splice sites, between Hlb320 and B6. All PCR products were sequenced using an Applied Biosystems 3730 DNA Analyzer system (Applied Biosystems, Foster City, CA).

### Hearing Evaluation by Auditory Brainstem Responses (ABRs)

Five 9-week-old B6 and Hlb320 (N3F11) males were anaesthetized with intraperitoneal injections of 2% tribromoethanol and placed on a heating pad set to 37.8°C. Platinum sub-dermal electrodes (Astro-Med, Inc., Warwick, RI) were inserted subcutaneously. The negative lead was inserted under the left ear, the positive lead was placed on the top of the head, and the ground was set between the eyes. The Smart EP High Frequency System (Intelligent Hearing System, Inc., Miami FL) was used to deliver both white noise at variable frequencies and decibels and pure tones as well as to record the electrical activity of the cells along the auditory pathway. Filters were set to exclude signals outside the range of 100–3000 Hz, and amplification was at 200 K with an analysis time of 10 msec (averaged responses were digitized and displayed on a PC screen). First stimulus presentation consisted of a white-noise click (2–8 kHz) at 70 dB, and depending on the response, was followed by increasing or decreasing volumes initially in 10 dB and subsequently in 5 dB steps to determine the auditory threshold. Mice were then subjected to pure-tone stimuli of 8, 16 and 32 kHz (duration 3 msec, 1.5 msec rise and fall time) and auditory brain response (ABR) was measured in dB SPL.

### Northern Analysis of *Hpn* mRNA

Total liver RNA from animals used for microarray analysis was also utilized to confirm lower *Hpn* expression level and to test for splice variants. Northern blot was performed according to the Ambion NorthernMax Kit (Ambion, Austin, TX) manual instructions. Briefly, the secondary structure of RNA samples was denatured by incubating the samples with added formaldehyde load dye for 15 minutes at 65°C. The samples were run on denatured agarose gel, transferred onto BrightStar-Plus positively charged nylon membrane (Ambion, Austin, TX) by downward transfer assembly, cross-linked using UV Stratalinker 1800 (Stratagene, La Jolla, CA), and hybridized with the BrightStar Psoralin-Biotin labeled (Ambion, Austin, TX) mouse hepsin probe for 12 hours at 55°C [23]. The antisense sequence of the probe is as follows: 5′-GTCCACGCAAAAGAAGCCCGATGTGCCGTTGGCGCCCGCAGTGCGCACAT-3′. The probe was designed to target exon 6 of *Hpn*-201 using the mouse genome map from NCBI (mm9 accessed in January 2011) and was made by Integrated DNA Technologies (IDT, Inc., Coralville, IA). The detection was done using the BrightStar BioDetect Kit (Ambion, Austin, TX) according to manufacturer's instructions. To assure equal loading and transfer of RNA, the same RNA was probed with a β-actin Mouse DECAtemplate probe provided with the NorthernMax Kit. β-actin is an internal control and assumed to be expressed at a constant level between samples. The hybridization with the β-actin Mouse DECAtemplate probe was done at 42°C.

### Histological Analysis of Liver

Livers from 20-week-old Hlb218 (N3F8), Hlb320 (N3F11), and B6 females fed chow diet were collected. One liver lobe was fixed in 10% neutral buffered formalin, embedded in paraffin, and stained with Mayer's hematoxylin and eosin (H&E). Another lobe from the same female was embedded in OCT, stained with oil red O, and counterstained with Mayer's hematoxylin.

### Histological Analysis of Susceptibility to Formation of Atherosclerotic Lesions in Aorta

Susceptibility to atherosclerosis was assessed as previously described [24], with some modifications. Briefly, sections from the aortic root were compared by visual examination of histological slides. Ten 20-week-old females from each strain (Hlb218 (N3F8), Hlb320 (N3F11), B6 and *Ldlr* ENU) were assessed: 5 females from each stain were kept on chow diet and the other 5 females were place on atherogenic diet at 9 weeks of age and kept on atherogenic diet for 10 weeks. Hearts from females sacrificed by cervical dislocation were collected, placed in 0.9% saline for at least 1 hour, trimmed from extraneous tissue (lung and thyroid) using a dissecting scope, and cut on a plane parallel to a plane formed by drawing a line between the tips of the atria. The top half of the heart with the atria and ascending aorta was fixed in 4% PFA (16% PFA diluted in PBS to 4% PFA) overnight, embedded in OCT, and sectioned using a cryostat at −20°C; 10-µm sections were stained with oil red O and counterstained with Mayer's hematoxilin. The cross section containing the area where the coronary artery and ascending aorta join was used as a landmark to identify the identical physiological region in each animal. All cross sections of the 300-µm area above the landmark in the aortic root were compared by visual examination.

### Western Blot for APOA1

Serum (4 males per strain; Hlb320 (N3F14)) was diluted in protein lysis buffer (1∶50 T-PER [Pierce, part of Thermo Fisher Scientific, Rockford, IL], 0.2% sodium dodecyl sulfate, and mini protease inhibitors cocktail tablet [Roche, Indianapolis, IN]). Serum protein concentration (µg/µl) was quantified using Bradford reagent (Sigma Life Sciences, St. Louis, MO) according to the manufacturer's instructions. Equal volumes of diluted serum were mixed with 4× XT-sample buffer (4∶1, Bio-Rad Laboratories, Hercules, CA) containing XT Reducing Agent (Bio-Rad Laboratories, Hercules, CA), incubated for 15 minutes at 65°C, separated on SDS polyacrylamide gel (Bio-Rad Laboratories, Hercules, CA), and electro-transferred to 0.45 µm nitrocellulose membrane (Bio-Rad Laboratories, Hercules, CA). The membrane was blocked overnight at 4°C, probed with primary rabbit anti-APOA1 antibody (ab40453, 1∶1,000, Abcam, Cambridge, MA), incubated with HRP-conjugated anti-rabbit secondary antibody (cat#7074S, 1∶5,000, Cell Signaling Technology, Inc., Danvers, MA), detected with Amersham ECLplus western blotting detection system (GE Healthcare Bio-Sciences, Piscataway, NJ), and visualized on Kodak scientific imaging film. The quantity of APOA1 protein level was calculated by dividing the intensity of the APOA1 protein band, measured using the ImageJ 1.44o program (National Institutes of Health, Bethesda, WD), by total serum protein concentration (µg/µl). Comparison of normalized serum APOA1 level between 2 groups was done with Student's 2-sample t-test using JMP9 (SAS Institute, Inc., Cary, NC).

## Results

### Generating the ENU Mutant Lines

The Jackson Laboratory's Heart, Lung, Blood, and Sleep Disorder Mutagenesis program generated ENU mutant mice (G0) by treating B6 males with ENU, an alkylating agent that induces random point mutations in the DNA of spermatogonial stem cells by virtue of single-base mismatching to the unrepaired alkylated base. We estimated that G1 mice carry 150 mutations on average, and subsequent backcrossing and inbreeding would further reduce the number of non-causal mutations. We expect each additional backcross to reduce the number of mutation by 50% [12]. Phenotyping of G3 progeny identified two unique G3 animals with low HDL cholesterol levels that were then backcrossed to B6 [12]. The progeny of B6×G3 was estimated to carry on average approximately 38 mutations, which subsequently was used to establish the lines (Hlb218 and Hlb320) by further intercrossing animals with low HDL cholesterol. HDL cholesterol levels in these two newly established lines were significantly lower compared to HDL cholesterol levels in B6 mice (Table 1).

**Table 1:** Serum total cholesterol, HDL-cholesterol, and triglyceride levels for B6, Hlb218, and Hlb320. Each value is expressed as mean (mg/dL) ± SD measured in serum (n = 5 per strain).

	Strain	Total cholesterol	HDL-cholesterol	Triglycerides
Females	B6	74.2±5.5	60.9±3.7	112.0±13.9
	Hlb218	5.0±3.4**	5.1±3.4**	47.0±18.5**
	Hlb320	46.2±3.0**	39.1±2.2**	79.0±13.5*
Males	B6	90.0±4.7	79.6±3.6	106.4±6.3
	Hlb218	5.8±1.7**	6.2±3.0**	43.3±12.5**
	Hlb320	67.0±6.6**	60.6±4.9**	84.0±6.3*

** P<0.0001.

* P<0.01.

### Low HDL Cholesterol Levels in Hlb218 Is Caused by a Mutation in *Pla2g12b*

**Figure 1:**
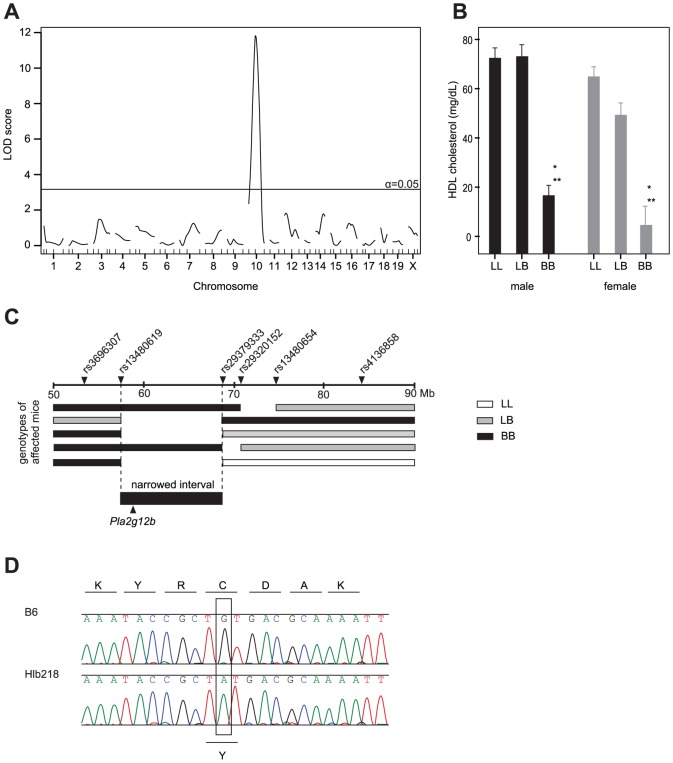
Identification of the mutation in C57BL/6J-*Pla2g12b^Hlb218^*/J on Chr 10. [**A**] Linkage analysis of (Hlb218×C57L) F2 animals for plasma HDL cholesterol levels showed a significant linkage on Chr 10; LOD score of 11.8 at α = 0.05. [**B**] Mean plasma HDL cholesterol values (HDL-C±SEM) by genotype and sex in the F2 population at peak marker *rs13480619* (*significant difference compared to LL (P<0.001); **significant difference compared to LB (P<0.001)). [**C**] Genotyping for additional SNP markers in F2 animals with low HDL cholesterol level and recombination in the mapped region narrowed the region with the mutation to 11.73 Mbp (between dashed vertical lines). Triangles above the upper black line are markers; numbers below the line represent the physical Mb location on Chr 10 (NCBI, mm9). [**D**] Chromatographs of genomic DNA sequence of the Hlb218 mouse versus the B6 control. The open rectangle highlights the transition of G to A in exon 3 of *Pla2g12b*. Corresponding amino acids are shown by the appropriate single letter code above the chromatographs.

The analysis of the F2 progeny from a cross between Hlb218 and C57L localized the mutation on Chr 10, with a peak near single-nucleotide polymorphism (SNP) marker *rs13480619* and a significant LOD score of 11.8 (Figure 1A). Comparison of plasma HDL cholesterol levels by genotype (one-way ANOVA) in F2 mice at the peak marker suggested that the mode of inheritance of low HDL cholesterol levels in Hlb218 is recessive: F2 mice that were homozygous for the B6 (BB) allele had significantly lower HDL cholesterol levels compared to F2 mice that were heterozygous (LB) or homozygous for the C57L (LL) allele (Figure 1B). The interval containing the mutation was further narrowed to 11.73 Mbp (Figure 1C) by genotyping the affected animals with a crossover within the interval using additional SNP markers. The analysis of high-throughput sequence data of liver RNA transcripts in the 11.73 Mbp interval revealed only one single point mutation in the region, while liver expression analysis (both microarray and RNAseq) did not show any significantly differentially expressed genes within the interval. The mutation, in the third exon of *Pla2g12b*, causes a transition of G to A that transforms the TGT (cysteine) codon into TAT (tyrosine) at amino acid position 129. Sanger sequencing of the exon confirmed the mutation (Figure 1D). The mutated cysteine in *Pla2g12b* is conserved among 12 eutherian mammals. In accordance with the guidelines for mouse strain and genetic nomenclature, the Mouse Genomic Nomenclature Committee named the allele *Pla2g12b^Hlb218^* and the strain C57BL/6J-*Pla2g12b^Hlb218^*/J.

### *Pla2g12b^Hlb218^* Alters Serum Lipid Levels and Increases Hepatic Triglycerides

**Figure 2:**
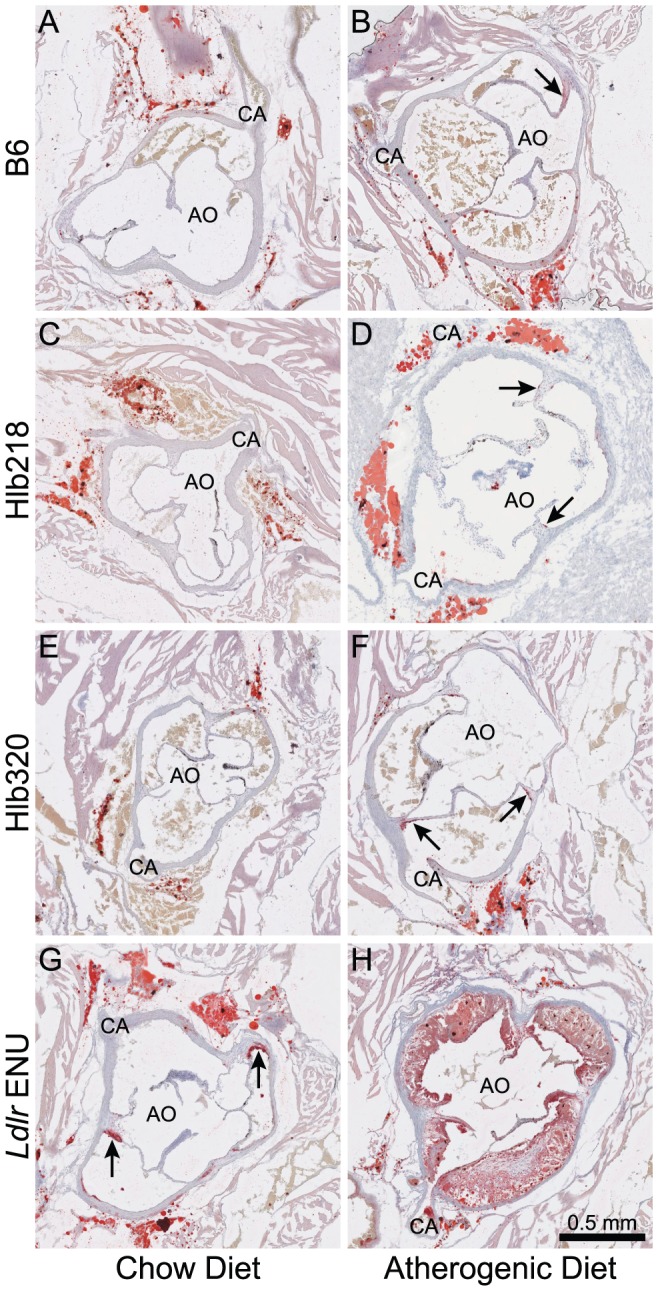
Comparison of atherosclerotic lesion size between mutant lines and B6. Hypolipidemic 20-week-old ENU females (Hlb218 and Hlb320) showed similar susceptibility to atherosclerosis (lesion formation) as age-matched B6 females on chow (panels C and E vs. A) and atherogenic diet (panels D and F vs. B). All cross sections of the 300-µm area above the aortic root, where coronary arteries (CA) and ascending aorta (AO) join, were compared (n = 5 females per strain for each diet; 2.5× magnification). Cross sections were stained with oil red O and counterstained with Mayer's hematoxilin. The figure shows representative cross sections from selected females. The black arrow points to areas with lesion formation. Cross sections from *Ldlr* ENU (panels G and H) were included as a positive control.

**Figure 3:**
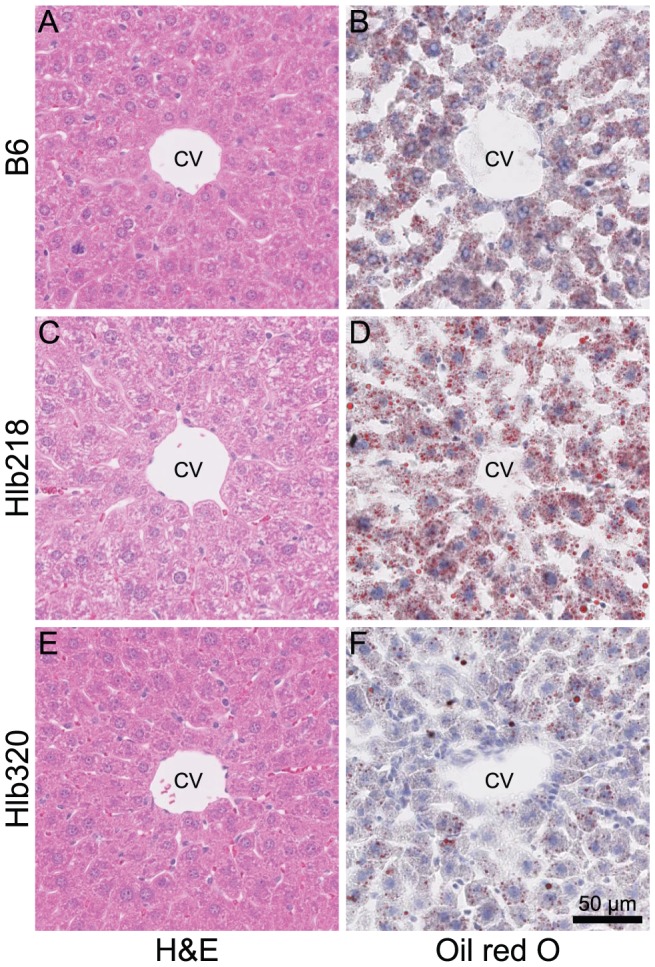
Histological comparison of liver from mutant lines and B6. Livers from 20-week-old Hlb218, Hlb320, and B6 females fed chow diet were collected. Liver cross sections from 5 females of each strain were stained with H&E and oil red O and compared. The figure shows representative liver sections from selected females. A, C, E — H&E stain; B, D, F — oil red O stain with Mayer's hematoxylin counterstain. Hlb218 mice showed increased liver lipid level. CV – central vein.

In addition to a 92% decrease in HDL cholesterol, the mutation also led to a 58% reduction in triglyceride levels (Table 1). Despite the abnormal lipid profile, the size of atherosclerotic lesions in Hlb218 mice remained similar to B6 (Figure 2 C–D vs. A–B). Histological analysis of livers from Hlb218 females showed an increase in accumulation of fat droplets (Figure 3 C–D vs. A–B).

### Low HDL Cholesterol Levels in Hlb320 Is Caused by a Mutation in *Hpn*

**Figure 4:**
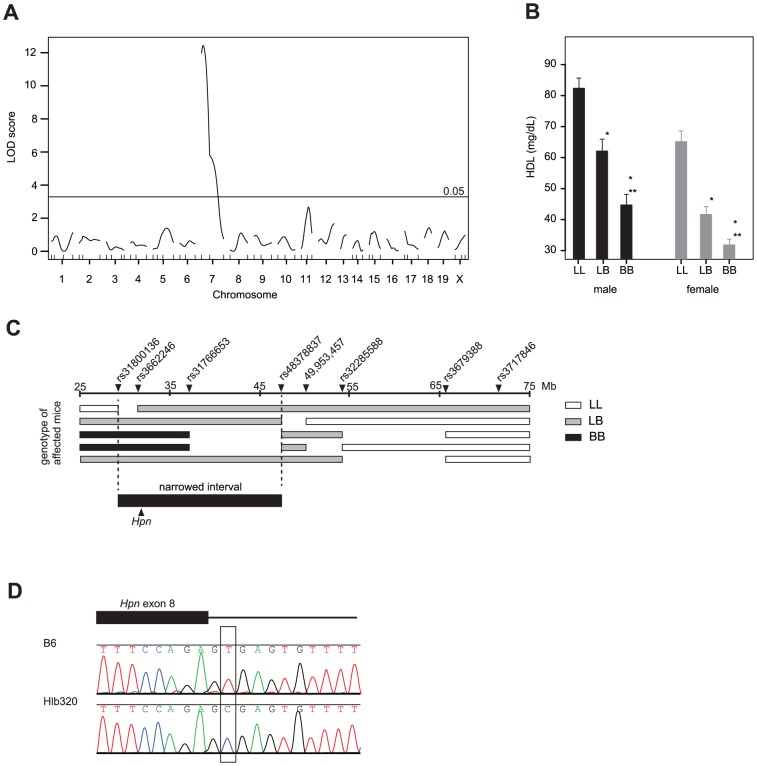
Identification of the mutation in C57BL/6J-*Hpn^Hlb320^*/J on Chr 7. [**A**] Linkage analysis of (Hlb320×C57L) F2 animals for plasma HDL cholesterol levels showed a significant linkage on Chr 7; LOD score of 12.4 at α = 0.05. [**B**] Mean plasma HDL cholesterol values (HDL-C±SEM) by genotype and sex in the F2 population at peak marker *rs4226386* (*significant difference compared to LL (P<0.0001); **significant difference compared to LB (P<0.01)). [**C**] Genotyping for additional SNP markers in F2 animals with low HDL cholesterol level and recombination in the mapped region narrowed the region with the mutation to 21.2 Mbp (between dashed vertical lines). Triangles above the upper black line are markers; numbers below the line represent the physical Mb location on Chr 7 (NCBI, mm9). [**D**] Chromatographs of genomic DNA sequence of the Hlb320 mouse versus the B6 control. The open rectangle highlights the transition of T to C in the second base pair in the 3′ splice site of exon 8 of *Hpn*.

**Figure 5:**
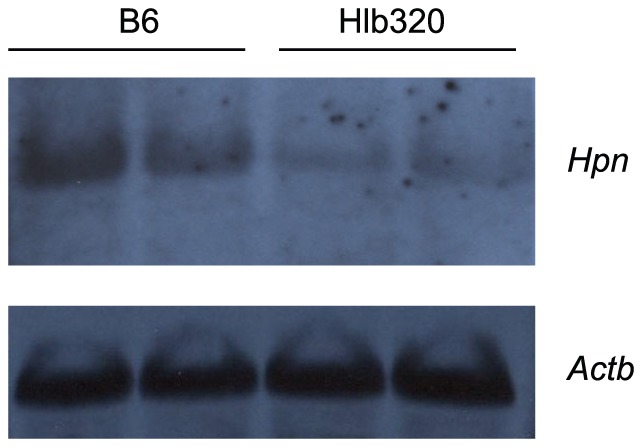
Northern blot analysis of *Hpn* mRNA expression. Total liver RNA from B6 and Hlb320 mice was hybridized with a mouse *Hpn* oligo probe and β-actin probe (loading control). The mRNA length of *Hpn* in B6 and Hlb320 was the same, while liver expression was relatively lower in Hlb320 compared to B6.

The chromosomal position of the mutation in Hlb320 was identified using the same strategy as used for Hlb218. The analysis of the F2 progeny from a cross between Hlb320 and C57L mapped the mutation to Chr 7, with a peak near SNP *rs4226386* and a significant LOD score of 12.4 (Figure 4A). One-way ANOVA of plasma HDL cholesterol by genotype in F2 mice at the peak marker suggested that the mode of inheritance of low HDL cholesterol levels in Hlb320 was additive: F2 mice homozygous for the B6 allele (BB) had significantly lower HDL cholesterol levels compared to F2 mice homozygous for the C57L (LL) allele, while F2 mice that were heterozygous (LB) had an intermediate HDL cholesterol level (Figure 4B). The interval containing the mutation was further narrowed to 21.2 Mbp (Figure 4C) by genotyping the affected animals with a crossover within the interval using additional SNP markers. Comparison of gene expression in the recombinant interval identified 3 differentially expressed genes in the region: *Hamp* (approximately 2.65-fold downregulated), *Hamp2* (approximately 4-fold downregulated), and *Hpn* (approximately 7-fold downregulated). Sequencing of the promoter region, the coding region, and splice sites of *Hamp* and *Hamp2* did not reveal any mutations (data not shown), suggesting that trans-regulation is the cause of the expression difference. Sequencing of *Hpn* identified a single nucleotide mutation of T to C in the second base pair in the 3′ splice site of exon 8 (Figure 4D). Northern blot analysis confirmed lower liver *Hpn* expression in Hlb320 in comparison to B6 and did not detect any alternative splice variants (Figure 5). Also, comparison of liver gene expression between Hlb320 and B6 revealed 106 significantly differentially expressed genes (q<0.05) located on other chromosomes (Table 2). In accordance with the guidelines for mouse strain and genetic nomenclature, the Mouse Genomic Nomenclature Committee named the allele *Hpn^Hlb320^* and the strain C57BL/6J-*Hpn^Hlb320^*/J.

**Table 2:** List of significantly (q<0.05) differentially expressed genes in Hlb320 livers compared to B6. Column 1 lists down-regulated genes; column 2 lists up-regulated genes.

Current gene name	Chr	Fold change	Current gene name	Chr	Fold change
*Hpn*	7	−7.02	*LOC194586*	UN	5.67
*H2-Q8*	UN	−4.00	*Hbb-b1*	7	3.99
*Hamp2*	7	−3.95	*Hba-a1*	11	3.63
*Foxq1*	13	−3.38	*Dct*	14	3.62
*Cib3*	8	−3.28	*Mfsd2a*	4	2.97
*H2-Q6*	17	−3.01	*Gstm2*	3	2.87
*Serpina12*	12	−2.94	*Lhpp*	7	2.71
*Hamp*	7	−2.65	*Ly6d*	15	2.61
*Slc3a1*	17	−2.50	*LOC384677*	UN	2.50
*LOC232400*	UN	−2.48	*Saa1*	7	2.47
*LOC241041*	UN	−2.40	*Slc1a2*	2	2.44
*Egr1*	18	−2.36	*Insig2*	1	2.34
*CRAD-L*	UN	−2.29	*Orm2*	4	2.17
*Rbp1*	9	−2.06	*Cyp2a5*	7	2.10
*Bdh2*	3	−2.03	*Zap70*	1	2.09
*Mug2*	6	−2.03	*Fitm1*	14	1.87
*LOC226654*	UN	−2.00	*Crygn*	5	1.86
*Foxa3*	7	−1.97	*Snhg11*	2	1.86
*H2-Q6*	17	−1.94	*Gstm3*	3	1.85
*Serpina1e*	12	−1.92	*St3gal6*	16	1.85
*Slc13a2*	11	−1.92	*Gal3st1*	11	1.84
*Gck*	11	−1.91	*Tox*	4	1.84
*Dclk3*	9	−1.86	*Ucp2*	7	1.79
*H2-Q7*	17	−1.82	*Lgals1*	15	1.77
*H2-D1*	17	−1.69	*Cyp4a12a*	4	1.75
*H2-Q5*	17	−1.68	*Rhbg*	3	1.74
*Sfxn1*	13	−1.67	*Elovl3*	19	1.73
*Acaca*	11	−1.66	*Lpl*	8	1.69
*Hgfac*	5	−1.65	*Cyp2a4*	7	1.67
*Irf5*	6	−1.64	*Rdh9*	10	1.67
*Hsd11b1*	1	−1.60	*Acnat2*	4	1.65
*Rps27*	3	−1.58	*Snhg11*	2	1.65
*Smyd1*	6	−1.58	*Gale*	4	1.64
*St6gal1*	16	−1.57	*Tlr5*	1	1.58
*Raet1b*	UN	−1.57	*Cib2*	9	1.57
*Amdhd1*	10	−1.55	*Fam25c*	14	1.56
*Cyp2f2*	7	−1.55	*Mvk*	5	1.55
*Gm2a*	11	−1.51	*Asns*	6	1.53
*Sox9*	11	−1.51	*Cdca3*	6	1.52
*Tmc7*	7	−1.45	*Gulo*	14	1.47
*Nme7*	1	−1.44	*Pltp*	2	1.46
*Got2*	8	−1.44	*Clstn3*	6	1.45
*Hrsp12*	15	−1.41	*Pafah2*	4	1.44
*Tsc22d4*	5	−1.40	*Ccl21a*	4	1.43
*Tpst1*	5	−1.39	*6430573F11Rik*	8	1.42
*Cpsf1*	15	−1.37	*Acot8*	2	1.41
*Gss*	2	−1.36	*Cyp1a2*	9	1.39
*Gas2*	7	−1.34	*Ccbp2*	9	1.38
*Tgfbr1*	4	−1.31	*Adhfe1*	1	1.37
*Il1rap*	16	−1.29	*Slc22a1*	17	1.36
*Rab14*	2	−1.21	*Cenpm*	15	1.35
			*Sepw1*	7	1.33
			*Mcm6*	1	1.29
			*Copz2*	11	1.27
			*Srxn1*	2	1.23

### *Hpn^Hlb320^* Alters Serum Lipid Levels without Affecting the APOA1 Levels, the Size of Atherosclerotic Lesions in Aorta, and Hepatic Triglyceride Levels

**Figure 6:**
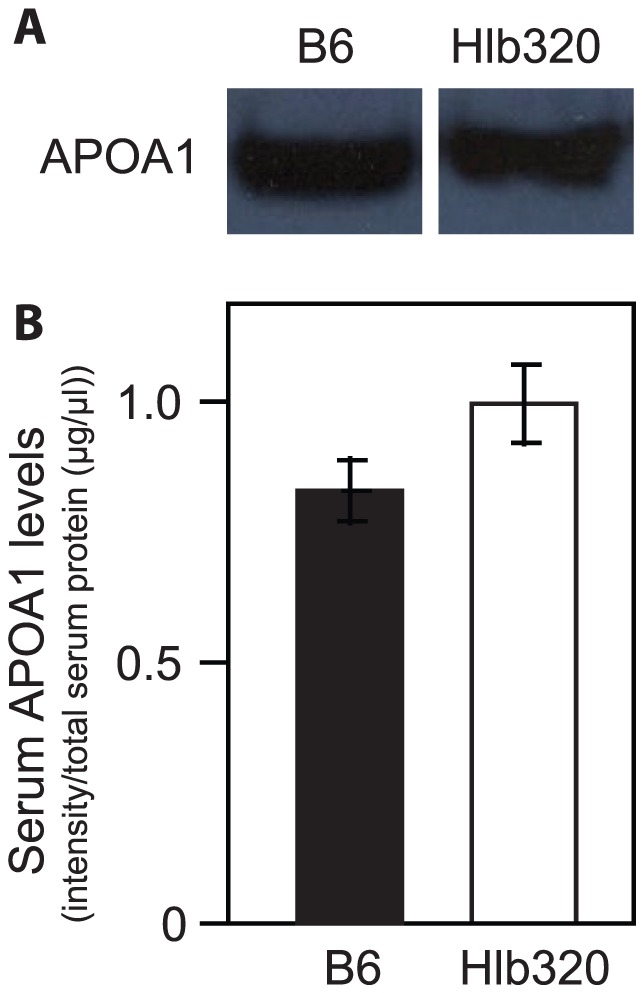
Serum APOA1 level in Hlb320 and B6. Western blot analysis showed similar serum APOA1 levels in Hlb320 (n = 4) and B6 (n = 4) male mice (P = 0.15). [**A**] Serum APOA1; bands from the western blot from representative animals. [**B**] Statistical comparison of quantified serum APOA1 level. The intensity of the APOA1 protein band for each animal was quantified and then normalized by the total serum protein concentration (µg/µl) in the sample from that animal. Normalized serum APOA1 level is expressed as mean±SEM.

The mutation led to a 24% reduction in HDL cholesterol level and a 21% reduction in triglyceride level (Table 1). Decreased cholesterol levels in lipoprotein particles could be caused by either decreased loading of cholesterol into the particle or by lower levels of the particles themselves. Because APOA1 is the most abundant apolipoprotein in an HDL particle, the APOA1 level could be used to estimate the level of HDL particle. Despite the reduction in HDL cholesterol levels in Hlb320 mice, APOA1 levels, measured by western blot, remained similar between Hlb320 and B6 (Figure 6). Although Hlb320 mice have an abnormal serum lipid profile, the size of their atherosclerotic lesions, as well as liver lipid accumulation, remained similar to B6 (Figure 2 E–F vs. A–B and Figure 3 E–F vs. A–B).

### Hlb320 Mice Show Similar Phenotypes as *Hpn* Knockout Mice

**Figure 7:**
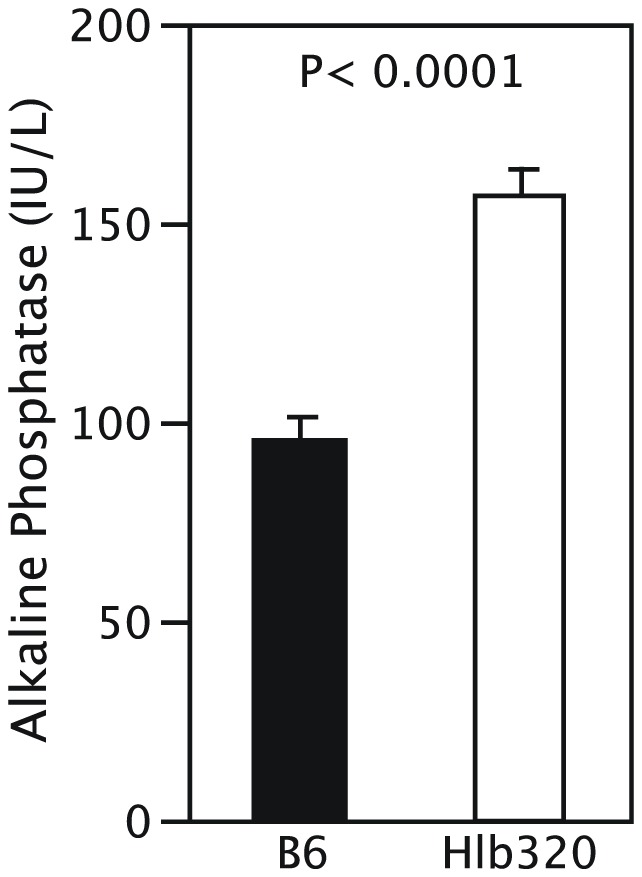
Serum alkaline phosphatase level in Hlb320 and B6. Serum total alkaline phosphatase (ALP) level in Hlb320 males (n = 5) was significantly higher than in B6 males (n = 5; P<0.0001). Total ALP activity is expressed as mean±SEM.

**Figure 8:**
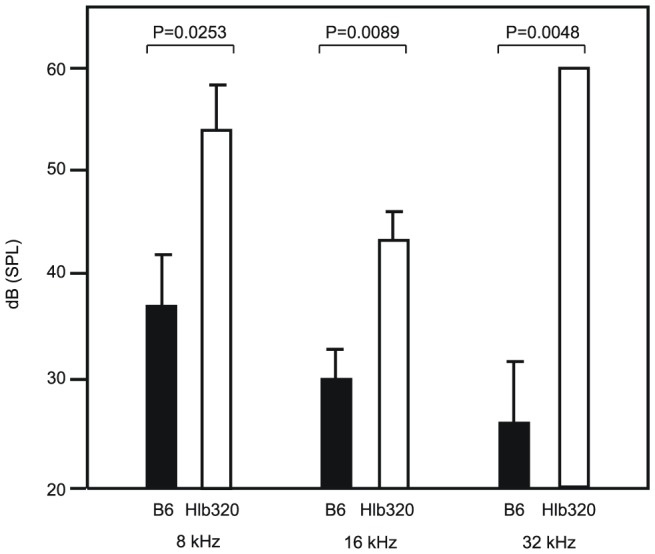
Comparison of hearing ability in Hlb320 and B6. Homozygous Hlb320 (n = 5) males had significantly lower hearing ability than age-matched B6 control males (n = 5) at 8 kHz, 16 kHz, and 32 kHz. Auditory brainstem response (ABR) threshold values (in dB SPL) are shown as mean±SEM for each auditory stimulus frequency (kHz).

An *Hpn* knockout mouse on a mixed B6/129 genetic background was previously generated by Wu et al [25]. These mice had higher serum alkaline phosphatase (ALP) level, loss of hearing, and lower thyroxine level compared to their control littermates [26]. Evaluation of these traits in Hlb320 males showed that, compared to age-matched B6 male controls, homozygous Hlb320 males also had significantly elevated serum ALP levels (Figure 7) and exhibited hearing loss (Figure 8), but did not show reduced thyroxine levels (data not shown).

## Discussion

The regulation of HDL cholesterol is strongly influenced by genetic factors, yet genes identified so far explain only a small portion of the heritability. To find novel genes that influence serum HDL cholesterol levels, we used ENU mutagenesis and identified causal mutations in 2 newly established ENU mouse lines with low HDL cholesterol levels.

The mutation in Hlb218 leads to a 92% decrease in HDL cholesterol and is a transition from G to A in the third exon of *Pla2g12b*, which leads to an amino acid change in the protein (C129Y). Analysis of the amino acid sequence using DiANNA, a software tool for cysteine state and disulfide bond partner prediction (http://clavius.bc.edu/~clotelab/DiANNA/), predicted the cysteine to be involved in formation of a disulfide bond [27]. The change of the amino acid would cause a loss of the disulfide bond and influence protein structure and binding capability. *Pla2g12b* encodes the group XIIB secreted phospholipase A2 (sPLA2GXIIB) and belongs to a family of structurally related enzymes (sPLA2). Unlike other sPLA2 enzymes, sPLA2GXIIB is catalytically inactive and was hypothesized to act as a ligand [28]. Lack of *Pla2g12b* has recently been shown to cause decreased serum lipids (total cholesterol, HDL cholesterol, triglycerides, and free fatty acids) and increased liver fatty droplets [29].

The mutation in *Pla2g12b* causes homozygous Hlb218 mice to have low serum total cholesterol, HDL cholesterol, and triglyceride levels as well as to accumulate lipid droplets in liver. In addition, their litter size is smaller (2–3 pups per litter), suggesting that this gene may play a role in fertility or gestation. Hlb218 mice appear to be smaller at birth and tend to develop more slowly in comparison to B6 mice, but catch up to B6 in size as they age. Despite low cholesterol levels and fatty liver, after 10 weeks on an atherogenic diet, atherosclerotic lesions in Hlb218 mice were similar in size to those in B6. The *Pla2g12b* knockout mice, recently described by Guan et al, and Hlb218 mutant mice have both shared and unique phenotypes [29]. Both mouse models have very low serum lipid levels and accumulate fatty droplets in the liver. While the decrease in cholesterol level is similar in both mouse models (approximately 92% reduction), the effect on triglyceride level is lower in Hlb218 mutants compared to *Pla2g12b* knockouts (58% vs. 78% reduction). Unlike *Pla2g12b* knockout mice, Hlb218 mice showed no differentially expressed genes in the liver. While Guan et al identified several downregulated genes by qPCR (*Hmgcs1*, *Hmgcr*, *Fasn*, *Scd1*, *Slc27a3*, *Slc27a4*, *Slc27a5*, and *Slc27a6*), our microarray data showed no differentially expressed genes between livers from Hlb218 and B6 mice. One explanation for differences in the phenotype is the difference in genetic background: *Pla2g12b* knockout mice are on a mixed B6/129/FVB genetic background, while Hlb218 mice are on a uniform B6 background. Another explanation is the difference in genetic alternation of *Pla2g12b*: the knockout completely lacks expression of the functional gene, while our mutant has normal gene expression with an amino acid change in the protein.

The precise mechanism and the mode of the effect (direct or indirect) by which *Pla2g12b* dysfunction affects serum lipid levels and leads to hepatic steatosis must still be elucidated. Hypolipidemia and hepatic steatosis are maladaptive and can result from the following: 1) an increased lipid supply inside the liver (increased endogenous synthesis of cholesterol and fatty acid accompanied by an inability to efflux synthesized lipid out of the liver); 2) reduced utilization by the liver (β-oxidation mitochondria); 3) reduced clearance of lipids from the liver (apoliporotein B packaging/secretion or the hepatobilliary pathway); 4) an increased lipid supply to the liver (influx of lipids from peripheral tissues/increased catabolism of lipids by the liver); or 5) changes in several other pathways (modification of lipoprotein particles in serum, uptake of lipoprotein particles by the liver, phosphatidylcholine biosynthesis or secretion, acceptance/storage of lipids in adipose tissue, discharge of bile into intestine, targeting of chylomicrons or functionality of chylomicrons released by the intestine, absorbance of lipids in the intestine, or insulin resistance) [30], [31], [32], [33], [34]. Liver gene expression data in *Pla2g12b* knockout mice and our Hlb218 mutant mice suggest that the first two possibilities (increased endogenous synthesis of cholesterol and fatty acid and changes in β-oxidation in mitochondria) are unlikely causes of abnormal lipid metabolism. The remaining above-mentioned pathways are still plausible explanations for hypolipidemia and hepatic steatosis, but considering the data by Guan et al, abnormal VLDL secretion seems to be a likely mechanism. To further prove that, in the absence of a functional sPLA2GXIIB, VLDL secretion from liver is dysfunctional, experimental results must show that 1) lower serum APOB100 and increased liver APOB levels are not caused by increased clearance of VLDL and LDL particles from serum by the liver; 2) lipid content of APOB48 containing lipoprotein particles remains unchanged; and 3) the lipid loading capacity of chylomicrons in the intestine is not affected by feedback from excess lipid in the liver. Further studies are needed to properly describe the role of *Pla2g12b* in lipid and lipoprotein particle metabolism and to dissect the responsible mechanism. Several other members of secreted PLA2 have already been found to play a role in cholesterol metabolism and atherosclerosis, and sPLA2GXIIB may be one of the missing links [35]. Both *Pla2g12b* knockout and the Hlb218 mutant are valuable tools in the further study of this gene and will be useful in dissecting how protein regulates lipids and lipoproteins in serum and liver.

The causal mutation in Hlb320 leads to a 24% decrease in HDL cholesterol and was identified as a change from a T into C in the second base pair in the 3′ splice site of exon 8 of *Hpn*. Hlb320 mice have a 7-fold lower *Hpn* mRNA expression. Lack of obvious alternative splice variant in the northern blot analysis and presence of a band of similar size but lower intensity in Hlb320 suggest that the mutation leads to either 1) an alternative splice variant of a size similar to the size of the B6 splice variant that is degraded either through a nonsense-mediated decay or no-go decay but cannot be easily identified as an alternative splice variant due to resolution of the northern blot; or 2) a reduced splicing rate. Although nonsense-mediated decay or no-go decay remain possible explanations for lower mRNA level, Aebi, et al showed that the T-C mutation at intron position +2 results in a correctly spliced product but at a reduced rate, which suggests that a reduced splicing rate is the likely mechanism to explain lower *Hpn* mRNA in Hlb320 [36]. *Hpn* encodes hepsin — a type II transmembrane serine protease expressed mainly on the surface of hepatocytes whose extracellular part has serine protease domain and a poorly conserved scavenger receptor cysteine-rich domain [37], [38], [39], [40]. Biochemical and in vitro studies have shown that hepsin participates in proteolytic digestion, initiation of blood coagulation, cell growth, and tissue remodeling. Its inhibition leads to growth arrest and changes in morphology, and its overexpression is associated with cancer [25], [41]. Previously published work did not test whether *Hpn* affects the serum lipid profile, and in the current study we show that a mutation in *Hpn* leads to low total cholesterol, HDL cholesterol, and triglyceride levels, suggesting a novel function of this gene in lipid metabolism.

Low lipid levels in Hlb320 mice did not lead to accumulation of fat in the liver and did not change the size of atherosclerotic lesions but did significantly affect liver metabolism as shown by differentially expressed genes. While genes coding for enzymes participating in glycolysis, glucose transport, lipogenesis, and formation of ketone bodies (*Gck*, *Foxa3, Acaca,* and *Bdh2*) were downregulated, genes coding for enzymes participating in β–oxidation, breakdown of triglycerides, urea cycle, synthesis of vitamin C, and reactive oxygen species elimination (*Acot8, Lpl, Rhbg, Asns, Gstm, Gulo, Gale, Cyp1a2,* and *Ucp2*) were upregulated [42], [43], [44], [45], [46], [47], [48], [49], [50], [51], [52]. Interestingly, *Elovl3*, *Insig2*, *Mvk*, *Fitm1*, *Rdh9*, *and Saa1* were also upregulated, suggesting increased cholesterol synthesis and cytosolic lipid droplet formation [53], [54], [55], [56], [57], [58]. Notably, genes involved in lipoprotein particle metabolism (other than *Saa1*) were not differentially expressed and serum APOA1 protein levels were not significantly different, suggesting that lipoprotein particles are not the cause of the abnormal lipid level phenotype (Table 2).

*Hpn* knockout mice, created by Wu et al, and Hlb320 mice showed similar phenotypes. Both *Hpn* knockout and Hlb320 mice are viable and fertile, have increased concentration of serum total nonspecific alkaline phosphatase, and decreased hearing ability [25], [26]. Unlike *Hpn* knockout mice, however, Hlb320 mice showed no difference in thyroxine levels (data not shown). While Wu et al reported no differences in growth and size, we observed Hlb320 pups to be smaller than B6 control mice. Some of the differences between the knockout and our mutant might be due to the difference in genetic background (*Hpn* knockout mice are on a mixed B6/129 genetic background, while Hlb320 mice are on a uniform B6 background) or due to a difference in the level of expression (the knockout completely lacks expression of the functional gene while our mutant has reduced expression).

The mechanism by which hepsin affects serum lipid levels remains to be studied. Since serum analysis showed low lipid levels with no increase in lipid accumulation in liver or aorta, we speculate that lipids from serum and synthesized in the liver must be utilized by hepatocytes for energy and necessary metabolites (bile acids, hormones, etc). Increased β-oxidation, detoxification through the glutathione-ascorbate cycle, and urine formation and decreased glycolysis and lipogenesis, as shown in our microarray analysis, supports the hypothesis that lipolysis and higher energy expenditure through β-oxidation of triglycerides are taking place in hepatocytes. Such changes in metabolism of the liver can be triggered by excessive exercise, lack of food intake (starvation), or abnormal hormonal signaling (epinephrine, norepinephrine, glucagon, growth hormone, testosterone). One possibility is that the cleavage target of hepsin affects one of the above processes. Hepsin has been shown to cleave pro-hepatocyte growth factor (pro-HGF), coagulation factor VII, laminin 332 pro-urokinase plasminogen (pro-UPA), pro-macrophage-stimulating protein (pro-MSP), the extracellular domain of the epidermal growth factor receptor, and prostasin [59], [60], [61]. Hepsin is one of the 3 most efficient proteases known to cleave pro-HGF [62]. Hepatocyte growth factor (HGF, mature form of pro-HGF) is a molecule that not only has been shown to play a role in embryonic development, migration, morphogenesis, regeneration, cell survival in various tissues, and diseases including cancer, but has also been recently shown to govern hepatic glucose metabolism through an HGF-cMet-Insulin receptor hybrid [62], [63]. This latest finding makes HGF a good candidate for explaining a potential mechanism that leads to abnormal lipid levels in our Hlb320 mutant. If hepsin cleaves pro-HGF into mature HGF, then lack of or lower expression of hepsin would lead to lower circulation of mature HGF and less glucose absorbance by hepatocytes. Shortage of glucose in the liver would lead to utilization of lipids and proteins as an energy source, which would dramatically change metabolism. Interestingly, HGF has also been shown to stimulate receptor kinase activity in both osteoclasts and osteoblasts, which could be a potential explanation for elevated bone ALP, and mutations in HGF have been shown to lead to hearing loss [64], [65]. The HGF-cMet system is already a target for the development of clinical therapeutics for many diseases, including cancer [66]. Mouse models like our Hlb320 mutant and the *Hpn* knockout will be useful in dissecting the mechanism of HGF regulation and its effect on lipid metabolism and in better understanding potential side effects of current drugs targeting HGF.

To summarize, we demonstrated that genetic mapping of an ENU mutant with a closely related inbred strain is an efficient method to identify genes involved in the phenotype under investigation when combined with identification of expression and coding differences in the mapped interval using microarray and high-throughput RNA sequencing. We identified novel mutations in *Pla2g12b* and *Hpn* that affect serum HDL cholesterol levels as well as other lipid levels. These new mouse models will be useful in the further dissection of the pathways leading to differences in cholesterol levels and metabolic disease.
